# Performance of the UCLA Scleroderma Clinical Trials Consortium Gastrointestinal Tract 2.0 instrument as a clinical decision aid in the routine clinical care of patients with systemic sclerosis

**DOI:** 10.1186/s13075-021-02506-x

**Published:** 2021-04-22

**Authors:** Norina Zampatti, Alexandru Garaiman, Suzana Jordan, Rucsandra Dobrota, Mike Oliver Becker, Britta Maurer, Oliver Distler, Carina Mihai

**Affiliations:** 1 Department of Rheumatology, University Hospital Zurich, University of Zurich, Gloriastrasse 25, 8091 Zurich, Switzerland; 2 Department of Rheumatology and Immunology, University Hospital Bern, University of Bern, Bern, Switzerland; 3 Department of Internal Medicine and Rheumatology, Cantacuzino Hospital, Carol Davila University of Medicine and Pharmacy, Bucharest, Romania

**Keywords:** Systemic sclerosis, Gastrointestinal, UCLA GIT 2.0, Endoscopy, Cohort study

## Abstract

**Background and objectives:**

The University of California Los Angeles Scleroderma Clinical Trial Consortium Gastrointestinal Tract Instrument 2.0 (UCLA GIT 2.0) is validated to capture gastrointestinal (GI) tract morbidity in patients with systemic sclerosis (SSc). The aims of this study were to determine in a large SSc cohort if the UCLA GIT 2.0 is able to discriminate patients for whom a rheumatologist with experience in SSc would recommend an esophago-gastro-duodenoscopy (EGD), and if it could identify patients with endoscopically proven esophagitis or with any pathologic finding on EGD.

**Methods:**

We selected patients fulfilling the ACR/EULAR 2013 criteria for SSc from our EUSTAR center having completed at least once the UCLA GIT 2.0 questionnaire, and we collected data on gastrointestinal symptoms and EGD from their medical charts. We analyzed by general linear mixed effect models several parameters, including UCLA GIT 2.0, considered as potentially associated with the indication of EGD, as well as with endoscopic esophagitis and any pathologic finding on EGD.

**Results:**

We identified 346 patients (82.7% female, median age 63 years, median disease duration 10 years, 23% diffuse cutaneous SSc) satisfying the inclusion criteria, who completed UCLA GIT 2.0 questionnaires at 940 visits. EGD was recommended at 169 visits. In multivariable analysis, UCLA GIT 2.0 and some of its subscales (reflux, distention/bloating, social functioning) were associated with the indication of EGD. In 177 EGD performed in 145 patients, neither the total ULCA GIT 2.0 score nor any of its subscales were associated with endoscopic esophagitis, nor with any pathologic EGD findings.

**Conclusions:**

In a real-life setting, the UCLA GIT 2.0 and its reflux subscale were able to discriminate patients with SSc who had an indication for EGD, but did not correlate with findings in EGD. We conclude that, while using the UCLA GIT 2.0 in the routine care of patients with SSc may help the rheumatologist to better understand the burden of GI symptoms in the individual patient, it should not be used as a stand-alone instrument to identify an indication of EGD.

**Supplementary Information:**

The online version contains supplementary material available at 10.1186/s13075-021-02506-x.

## Introduction

In patients with systemic sclerosis (SSc), the gastrointestinal (GI) tract is the most common internal organ involvement, with over two thirds of patients reporting GI symptoms [1]. SSc GI tract involvement is a major cause of serious morbidity, affecting health-related quality of life (HRQoL) and survival of these patients [2, 3]. The most prevalent GI manifestation is esophageal involvement due to hypomotility and gastroesophageal reflux, the latter often leading to esophagitis and in later stages to Barrett’s esophagus [4]. Another GI manifestation of SSc is gastric antral vascular ectasia (GAVE) which may cause severe anemia [5]. To date, there are no recommendations or guidelines when to perform endoscopic and functional investigation of the upper GI tract in patients with SSc. Esophago-gastro-duodenoscopy (EGD) plays a major role in the diagnosis of reflux esophagitis, esophageal strictures, Barrett’s esophagus, and adenocarcinoma of the esophagus.

The University of California at Los Angeles Scleroderma Clinical Trial Consortium GIT 2.0 instrument (UCLA GIT 2.0) is a patient-completed questionnaire validated to assess GI symptoms severity and related HRQoL in SSc [6]. Originally developed in English, and with a minimal clinically important difference previously determined [6], it has been validated in different languages [7–11]. Several clinical trials of GI treatments in patients with SSc already used this instrument as an outcome measurement [12–14]. The UCLA GIT 2.0 is an excellent candidate to guide the need for further investigation of the GI tract by endoscopy and/or functional tests. Constructed to reflect the burden of GI symptoms including reflux, it is attractive to hypothesize that it is able to identify patients with endoscopic esophagitis or other clinically significant findings on EGD.

In this study, we aimed to determine, in an unselected, real-life cohort of patients with SSc, whether the UCLA GIT 2.0 could discriminate patients for whom a rheumatologist with experience in SSc would recommend an EGD, and if the UCLA GIT 2.0 could identify patients at risk for endoscopic esophagitis or other clinically significant EGD findings.

## Methods and patients

### Study population

For this observational, post hoc analysis of prospectively collected data from the SSc cohort of the University Hospital Zurich, we selected patients who were included in the European Scleroderma Trials and Research Group (EUSTAR) database, fulfilled the ACR/EULAR 2013 criteria for the classification of SSc, and completed at least one UCLA GIT 2.0 questionnaire. Our center is following the EUSTAR recommendations for a detailed annual assessment, based on a standardized clinical approach and work-up [1]. Patients also complete additional questionnaires at their annual visits as part of that routine assessment, including the UCLA GIT 2.0. Investigations of the GI tract, such as EGD, are not included in the routine assessment and are selectively recommended by the expert rheumatologist, after taking the history, performing the clinical examination of the patients, and evaluating their same-day work-up results (laboratory, lung function tests, lung imaging, electrocardiogram, and power-Doppler echocardiography). There was no regular use of the UCLA GIT 2.0 questionnaire to decide further GI investigation, although the rheumatologist could have access to the patient self-reported data, at least in part of the cases.

Data were retrieved from the prospectively collected EUSTAR registry for our center. In the EUSTAR database, information on gastrointestinal involvement is recorded by 3 items: esophageal symptoms (reflux and/or dysphagia), stomach symptoms (early satiety and/or vomiting), and intestinal symptoms (diarrhea, bloating, and/or constipation). To collect more detailed data on upper GI symptoms, presence of EGD, and treatment with proton pump inhibitors (PPI), we additionally reviewed retrospectively the electronic medical records (EMR) of the selected patients (see details in the online supplement). We also recorded the attending rheumatologist’s indication to perform an EGD from each visit of the patient. As some patients had more than one EGD, we selected for further analysis the EGD performed within a period of up to 3 months before or after the corresponding EUSTAR assessment visit and, if more than one EGD, the one closest to the corresponding visit. Reflux esophagitis was graded according to the Los Angeles classification [15]. Patients with concomitant acute GI bleeding or a history of cancer in the upper GI tract were excluded from this study. The study has been performed in accordance with the Declaration of Helsinki Ethical Principles and with GCP guidelines. Ethical approval for this data collection and analysis was issued by the cantonal ethics (BASEC Nr. PB2016-01515 and 2018-02165).

### UCLA GIT 2.0 questionnaire and study outcomes

The UCLA GIT 2.0 questionnaire contains 34 items, organized into seven subscales: reflux, distention/bloating, diarrhea, fecal soilage, constipation, emotional wellbeing, and social functioning. The subscales are scored from 0 to 3, higher scores indicating more severe symptomatology and worse HRQoL. Scoring of the diarrhea and constipation scales is different, ranging from 0 to 2 and 0 to 2.5, respectively. The total UCLA GIT 2.0 score is calculated by averaging all subscales, except the one for constipation, and ranges from 0 to 2.83 [6, 7].

We defined three study outcomes: first, the recommendation to perform EGD by the SSc-specialized rheumatologist; second, macroscopic esophagitis identified on EGD (based on the EGD report and mentioning the Los Angeles grade of esophagitis), further referred to as “endoscopic esophagitis”; and third, any significant pathologic finding on EGD, further referred to as “pathologic EGD.” The latter included endoscopic esophagitis, mycotic esophagitis, esophageal strictures, Barrett’s esophagus, gastric antral vascular ectasia (GAVE), peptic ulcers, and tumors.

### Statistical analysis

For statistical calculations, we used the statistic software IBM SPSS 25.0 and R language 3.6 (*lme4* package) [16]. A *p* value < 0.05 was considered statistically significant. Numeric variables are described as median and inter-quartile range (Q1, Q3), while categorical variables are described as *n* and percentage. Comparisons between groups were performed with the chi-squared test for categorical variables and with the Mann-Whitney *U* test for numeric variables.

The parameters of interest for all three study outcomes were the UCLA GIT 2.0 total score and its reflux, distention/bloating, social functioning, and emotional wellbeing subscales. We analyzed their association with each of the three dichotomous outcomes of the study using multivariable generalized linear mixed effects models (GLMM) adjusted for random effects of subjects and fixed effects for all other candidate parameters mentioned. For the first outcome (recommendation to perform EGD), we excluded patients who had performed EGD during the last 3 months before their visit to our center, considering that in most of these patients a new EGD would not be recommended again at the assessment.

The following parameters, which potentially influence the study outcomes (further referred as “covariates”) were selected by the authors based on clinical experience and evidence from published literature: age, sex, disease duration, cutaneous subset of SSc (diffuse vs. any other subset) [17], modified Rodnan skin score (mRSS), body mass index (BMI), hemoglobin (Hb), erythrocyte sedimentation rate (ESR), forced vital capacity (FVC), PPI therapy, gastro-esophageal symptoms as retrieved from the charts of the patients (heartburn, regurgitation, dysphagia, and vomiting), “esophageal symptoms” as recorded in the EUSTAR database (reflux and/or dysphagia), and “stomach symptoms” as recorded in the EUSTAR database (early satiety and/or vomiting).

For the outcome “recommendation to perform EGD,” we performed the following GLMM models using as covariates: 1. age, sex, disease duration, mRSS, SSc subset, BMI, Hb, ESR, FVC, and PPI therapy, which were included in all the other models; 2. gastro-esophageal symptoms, as collected from the patient charts (heartburn, regurgitation, dysphagia, vomiting); 3. “esophageal symptoms” and “stomach symptoms” as recorded in EUSTAR database; and in models 4 to 8, one of the selected subscales of UCLA GIT 2.0 (reflux, distention/bloating, social functioning, emotional wellbeing) or the UCLA GIT 2.0 total score, respectively.

For the outcomes “endoscopic esophagitis” and “pathologic EGD,” anticipating that the number of EGD will be less than one third than the number of visits with a completed UCLA GIT 2.0 questionnaire, we reduced, by clinical judgment, the number of covariates included in the multivariable analysis. Consequently, all GLMM models for these outcomes included only four independent variables, selected by clinical judgment: age, sex, disease duration, and PPI therapy. Further GLMM models included these four parameters and one of the following parameters, or group of parameters: mRSS (model 2), Hb (model 3), gastroesophageal symptoms reported by the patient: heartburn, regurgitation, and dysphagia, as collected from EMR (model 4), “esophageal symptoms” and “stomach symptoms” (model 5), and one of the selected subscales of UCLA GIT 2.0 or the UCLA GIT 2.0 total score, respectively (models 6 to 10).

We further identified by receiver operating characteristic (ROC) curve analysis, selecting the values with the largest area under the curve (AUC) and significant 95% confidence intervals (95% CI), cutoffs for the reflux, and total UCLA GIT 2.0 score discriminating best between patients with recommendation to perform EGD and those without. Based on the AUC, the accuracy of the prediction model can be considered excellent (0.9–1.0), good (0.8–0.9), fair (0.7–0.8), or poor (0.6–0.7).

## Results

### Patients and baseline characteristics

Out of 494 patients in the database, 346 were fulfilling the inclusion criteria. For these, 940 visits with a completed UCLA GIT 2.0 questionnaire were available. The median number of visits per patient was 2 (Q1, Q3: 1–4), with 89/346 patients having one visit. Median follow-up time was 3.4 years (Q1, Q3: 1.8–4.9).

The demographic and clinical data of the patients are displayed in Table 1. The majority of participants were female (82.4%) and Caucasian (94.5%), 23% had the diffuse cutaneous subtype of SSc, with a median age of 63 years and a median disease duration of 10 years. Nine out of 343 patients had a history of Barrett’s esophagus. Of 346 patients, 261 patients (75.4%) reported GI symptoms and 311/346 patients (89.9%) had UCLA GIT 2.0 scores > 0 in at least one visit, GI symptoms recorded from the patients’ charts and UCLA GIT 2.0 scores (median and interquartile range) are displayed in Table 2. The reflux and distention/bloating subscales and the total score of UCLA GIT 2.0 had medians of 0.25, 0.50, and 0.22, respectively, while the medians of the other subscales were zero. Approximately 10% of the components of the UCLA GIT 2.0 questionnaire were missing overall. Of 940 visits, treatment with PPI was present in 588 (62.6%) visits at the time of completing the UCLA GIT 2.0 questionnaire at the annual assessment.

**Table 1:** Baseline demographic and clinical characteristics of the study cohort

	*N*		
**Age (years):** median (Q1–Q3)	346	63	(51–72)
**Disease duration (years):** median (Q1–Q3)	346	10	(7–17)
**BMI (kg/m**^**2**^**):** median (Q1–Q3)	310	23.4	(21–27)
**Male sex:** *N* (%)	346	60	(17.3)
**Diffuse cutaneous subset:** *n* (%)	283	65	(23)
**Raynaud’s phenomenon:** *n* (%)	342	327	(95.6)
**Digital ulcers ever:** *n* (%)	335	114	(34.0)
**mRSS:** median (Q1–Q3)	337	3	(0–8)
**Joint synovitis:** *n* (%)	344	61	(17.7)
**Joint contractures:** *n* (%)	340	99	(29.1)
**Esophageal symptoms** (reflux, dysphagia): *n* (%)	344	185	(53.8)
**Stomach symptoms** (early satiety, vomiting): *n* (%)	341	102	(29.9)
**Intestinal symptoms** (diarrhea, bloating, constipation): *n* (%)	344	124	(36)
**Malabsorption syndrome:** *n* (%)	306	7	(2.3)
**Intestinal pseudo-obstruction:** *n* (%)	311	4	(1.3)
**Barrett’s esophagus**	343	9	(2.6)
**Pulmonary hypertension:** *n* (%)	331	35	(10.6)
**Thorax HRCT: lung fibrosis:** *n* (%)	328	135	(41.2)
**FVC:** median (Q1–Q3)	339	97	(84–110)
**Renal crisis:** *n* (%)	343	6	(1.7)
**ANA positive:** *n* (%)	345	340	(98.6)
**Anti-centromere positive:** *n* (%)	327	155	(47.4)
**Anti-topoisomerase I positive:** *n* (%)	332	84	(25.3)
**Anti-RNA polymerase III positive:** *n* (%)	310	36	(11.6)
**CRP (mg/dl):** median (Q1–Q3)	312	1.6	(0.7–4)
**ESR (mm/h):** median (Q1–Q3)	329	12	(6–23.5)
**Hb (g/dl):** median (Q1–Q3)	310	13.2	(12.4–14.1)

*BMI* body mass index, *mRSS* modified Rodnan skin score, *HRCT* high-resolution computer tomography, *FVC* forced vital capacity, *ANA* anti-nuclear antibodies, *CRP* C-reactive protein, *ESR* erythrocyte sedimentation rate, *Hb* hemoglobin

**Table 2:** Gastrointestinal symptoms and scores of the UCLA GIT 2.0 and its subscales in all visits, *N* = 940

	*n*	*%*	
**Heartburn**	323	(35.2)	
**Regurgitation**	*123*	(13.4)	
**Dysphagia**	*184*	(20.1)	
**Early satiety**	*106*	(11.6)	
**Vomiting**	*34*	(3.7)	
**Upper abdomen pain**	*26*	(2.8)	
**Bloating**	*157*	(17.1)	
**Diarrhea**	*84*	(9.2)	
**Constipation**	*109*	(11.9)	
**Fecal incontinence**	*58*	(6.3)	
**UCLA GIT 2.0 subscales**	Median	Q1, Q3	Range
**Reflux**	0.25	0.00, 0.63	(0–3)
**Distention/bloating**	0.50	0.00, 1.00	(0–3)
**Fecal soilage**	0.00	0.00, 0.00	(0–3)
**Diarrhea**	0.00	0.00, 0.50	(0–2)
**Social functioning**	0.00	0.00, 0.33	(0–2.5)
**Emotional wellbeing**	0.00	0.00, 0.22	(0–3)
**Constipation**	0.00	0.00, 0.50	(0–2.5)
**Total score of UCLA GIT 2.0**	0.22	0.07, 0.49	(0–2.4)

### Evaluation of the UCLA GIT 2.0 as a potential decision-aiding instrument for EGD

Of 940 visits with completed UCLA GIT 2.0 questionnaires, 31 were excluded from this part of the analysis because patients had an EGD within 3 months before the visit. In the 909 remaining visits, EGD was recommended in 169, of which 120 were carried out (Figure S1 in the online supplement). Patients with a recommendation for EGD had significantly more frequent heartburn, dysphagia, and regurgitation, a history of Barrett’s esophagus, as well as higher mRSS scores and erythrocyte sedimentation rates; they also had significantly higher values of the UCLA GIT 2.0 score and all its subscales except the subscale for fecal soilage (Table 3).

**Table 3:** Comparison of patient data from visits in which EGD was recommended (*n* = 169) vs. data from visits in which EGD was not recommended (*n* = 740)

	Referral to EGD Median (Q1, Q3)	No referral to EGD Median (Q1, Q3)	p*
Age	63 (52, 70.5)	63 (53, 72)	0.759
Disease duration	11 (7, 20)	10 (6,16)	0.086
**mRSS**	**3 (0, 8)**	**2 (0, 6)**	**0.009**
FVC	98 (85, 109)	97 (85, 110)	0.813
BMI	23.7 (21.4–26.7)	23.4 (21, 27)	0.712
Hemoglobin	13.1 (12, 14.2)	13.3 (12.4, 14.1)	0.118
**ESR**	**16 (9, 26)**	**12 (6, 22)**	**0.013**
	*n* (%)	*n* (%)	*p*^#^
Subset of SSc	Diffuse 38 (24.4) Limited 118 (75.6)	Diffuse 156 (25.2) Limited 464 (74.8)	0.836
**Heartburn**	**105 (60.7)**	**207 (29)**	**< 0.001**
**Regurgitation**	**41 (23.7)**	**78 (10.9)**	**< 0.001**
**Dysphagia**	**65 (38.5)**	**112 (15.6)**	**< 0.001**
Vomiting	10 (5.9)	22 (3.1)	0.074
**Esophageal symptoms**	**109 (72.2)**	**283 (43.5)**	**< 0.001**
**Stomach symptoms**	**66 (44.3)**	**139 (21.4)**	**< 0.001**
Barrett’s esophagus	17 (10.1)	5 (0.7)	< 0.001
PPI therapy	76 (62.3)	18 (81.8)	0.077
Digital ulcers	16 (16)	65 (15)	0.811
Joint contractures	46 (31.1)	226 (35.1)	0.354
UCLA GIT 2.0 subscales	Median (Q1, Q3)	Median (Q1, Q3)	p*
**Reflux**	**0.50 (0.13, 0.88)**	**0.14 (0.00, 0.50)**	**< 0.001**
**Distention/bloating**	**0.75 (0.25, 1.46)**	**0.25 (0.00, 1.00)**	**< 0.001**
Fecal soilage	0.00 (0.00, 1.00)	0.00 (0.00, 0.00)	0.067
**Diarrhea**	**0.00 (0.00, 0.50)**	**0.00 (0.00, 0.50)**	**0.028**
**Social functioning**	**0.33 (0.00, 0.60)**	**0.00 (0.00, 0.33)**	**< 0.001**
**Emotional wellbeing**	**0.11 (0.00, 0.75)**	**0.00 (0.00, 0.22)**	**< 0.001**
**Constipation**	**0.25 (0.00, 0.75)**	**0.00 (0.00, 0.50)**	**0.004**
**Total score of UCLA GIT 2.0**	**0.35 (0.17, 0.70)**	**0.19 (0.05, 0.44)**	**< 0.001**

Statistically significant results are highlighted in bold font

*Mann-Whitney *U* test, ^#^Chi-square test

*mRSS* modified Rodnan skin score, *FVC* forced vital capacity, *BMI* body mass index, *Hb* hemoglobin, *ESR* erythrocyte sedimentation rate, *PPI* proton pump inhibitors

We next aimed to identify independent parameters associated with the expert recommendation to perform EGD. We found in multivariable GLMM models that mRSS, individual gastroesophageal symptoms (heartburn, dysphagia, and regurgitation, respectively) and upper gastrointestinal tract symptoms as recorded in the EUSTAR database (“esophageal symptoms” and “stomach symptoms”), significantly associated with the recommendation to perform EGD. Except the emotional wellbeing subscale, all the examined subscales of UCLA GIT 2.0, as well as the total score, correlated significantly with the recommendation to perform EGD (Table 4).

**Table 4:** Factors associated with referral to EGD (multivariable generalized linear mixed effects models, GLMM). Statistically significant results are highlighted in bold font

Multivariable GLMM					
Parameters	Models*	OR	95% CI	***p***	AUC (95% CI)
Age	Model 1	0.99	0.97–1.01	0.269	0.75 (0.69–0.80)
Sex		0.66	0.33–1.33	0.242	
Disease duration		1.00	0.98–1.02	0.926	
SSc subset		1.27	0.67–2.40	0.471	
**mRSS**		**1.05**	**1.00–1.10**	**0.030**	
Hb		0.89	0.74–1.07	0.218	
PPI		0.80	0.49–1.30	0.363	
BMI		1.03	0.97–1.09	0.294	
FVC		1.00	0.99–1.01	0.948	
ESR		1.00	0.99–1.02	0.943	
Barrett’s esophagus		24.4	2.43–245.18	0.007	
**Heartburn**	**Model 2**	**2.23**	**1.35–3.69**	**0.002**	**0.71 (0.65–0.76)**
**Regurgitation**		**2.09**	**1.14–3.81**	**0.017**	
**Dysphagia**		**3.01**	**1.79–5.05**	**< 0.001**	
Vomiting		1.98	0.62–6.25	0.247	
**Esophageal symptoms**	**Model 3**	**1.91**	**1.14–3.18**	**0.013**	**0.68 (0.62–0.74)**
**Stomach symptoms**		**2.12**	**1.24–3.61**	**0.006**	
**Reflux subscale**	**Model 4**	**1.86**	**1.19–2.90**	**0.006**	**0.68 (0.62–0.74)**
**Distention/bloating subscale**	**Model 5**	**1.50**	**1.12–2.01**	**0.007**	**0.70 (0.65–0.76)**
**Social functioning**	**Model 6**	**2.57**	**1.56–4.23**	**< 0.001**	**0.65 (0.59–0.71)**
Emotional wellbeing	Model 7	1.32	0.80–2.19	0.274	0.68 (0.62–0.74)
**Total UCLA GIT 2.0 score**	**Model 8**	**2.16**	**1.21–3.83**	**0.009**	**0.64 (0.58–0.70)**

*Model 1 contains the covariates age, sex, disease duration, PPI therapy, mRSS, ESR, Hb, FVC, Barrett’s esophagus, BMI, and a subset of SSc. All other models contain, in addition to the covariates of model 1, the following covariates: model 2: heartburn, regurgitation, dysphagia, and vomiting; model 3: the symptom clusters “esophageal symptoms” and “stomach symptoms” as per expert opinion; and models 4–8: one of the mentioned subscales of UCLA GIT 2.0, respectively the total UCLA GIT 2.0 score

*mRSS* modified Rodnan skin score, *Hb* hemoglobin, *ESR* erythrocyte sedimentation rate, *PPI* proton pump inhibitors, *FVC* forced vital capacity, *BMI* body mass index

To identify optimal cutoffs for the reflux and total UCLA GIT 2.0 score, discriminating best between patients with recommendation to perform EGD and those without, we performed ROC analysis. For the reflux subscale, the best results were found for the cutoff of 0.163 (AUC [95% CI] of 0.64 [0.60–0.68]), with a sensitivity of 73% and specificity of 50%. Similarly, for the total UCLA GIT 2.0 score, we identified the optimal cutoff of 0.161, with an AUC [95%CI] of 0.64 [0.59–0.68], sensitivity 78%, and specificity 46%. As the range for these scores is 0–3 and 0–2.83 respectively, this shows that even patients with a low symptom burden have been referred to further evaluation by EGD.

### Evaluation of the UCLA GIT 2.0 as a potential predictor of endoscopic esophagitis and pathologic EGD

Of all 346 patients, 241 had undergone EGD at least once during the entire observation period. We identified 177 EGD matching the inclusion criteria, performed in 145 patients.

Of these, 128 were performed on indication from the SSc-expert rheumatologist of our center, and 49 were performed on indication from another physician, of which 31 were done during the 3 months preceding the visit (Figure S2 in the online supplement)**.** A single EGD was performed in 118 patients, 22 patients had undergone two EGDs, and five patients had undergone three EGDs. The median time between the visit and the corresponding EGD was 2 days (Q1, Q3: − 0.5, 36), with a mean of 9.7 days.

Esophagitis was found in 52/177 EGD (in 50 patients), GAVE in 15/177 EGD (in 12 patients), and biopsy-verified Barrett’s esophagus in 24 /177 EGD (in 19 patients). Other EGD findings were fungal esophagitis in 7, esophageal strictures in 2, peptic ulcers of the stomach or bulbus duodeni in 3, and gastritis in 6 EGD, leading to a total of 94/177 pathologic EGD.

Patients with endoscopic esophagitis had significantly more frequently EUSTAR reported esophageal symptoms (“reflux and/or dysphagia”) and slightly higher mRSS scores, while the distribution of individual upper gastrointestinal tract symptoms (heartburn, dysphagia, and regurgitation), as well as that of the UCLA GIT 2.0 score and subscales, did not reach statistical significance (Table 5). Patients with esophagitis also tended to be less frequently under treatment with PPI (52.7% vs. 72.4%, *p* = 0.057) while, surprisingly, they had slightly but significantly higher Hb values vs. patients without esophagitis (median Hb value13.6 g/dl vs 12.9 g/dl, *p* = 0.008).

**Table 5:** Comparison of patient data from visits in which EGD detected esophagitis (*n* = 52), respectively did not detect esophagitis (*n* = 125)

	Esophagitis Median (Q1, Q3)	No esophagitis Median (Q1, Q3)	p*
Age	62 (51, 69.5)	64 (56, 72)	0.086
Disease duration	10 (6, 19)	12 (7, 19)	0.487
**mRSS**	**5 (0, 11.75)**	**2 (0, 6)**	**0.002**
FVC	93.5 (76.5, 110.8)	99 (84, 108)	0.528
BMI	23.2 (21.6, 25.9)	23.8 (21.1, 28.1)	0.407
**Hb**	**13.6 (12.4, 14.6)**	**12.9 (11.6, 13.7)**	**0.006**
ESR	18 (8, 28)	16 (10, 28)	0.888
	*n* (%)	*n* (%)	*p*^#^
Subset of SSc	Diffuse 13 (26.5) Limited 36 (73.5)	Diffuse 24 (22) Limited 85 (78)	0.536
Barrett’s esophagus	9 (17.3)	15 (12)	0.347
Heartburn	32 (64)	62 (50.4)	0.104
Regurgitation	13 (26)	25 (20.3)	0.414
Dysphagia	18 (36)	46 (37.4)	0.863
Vomiting	5 (10)	5 (4.1)	0.129
**Esophageal symptoms**	**40 (81.6)**	**67 (60.4)**	**0.008**
Stomach symptoms	22 (46.8)	39 (35.5)	0.163
PPI therapy	30 (57.7)	89 (72.4)	0.057
Digital ulcers	9 (27.3)	11 (15.7)	0.217
Joint contractures	16 (33.3)	32 (29.1)	0.594
UCLA GIT 2.0 subscales	Median (Q1, Q3)	Median (Q1, Q3)	p*
Reflux	0.50 (0.25, 0.84)	0.38 (0.13, 0.88)	0.534
Distention/bloating	0.50 (0.06, 1.00)	0.75 (0.25, 1.50)	0.069
Fecal soilage	0.00 (0.00, 0.00)	0.00 (0.00, 1.00)	0.535
Diarrhea	0.00 (0.00, 0.50)	0.00 (0.00, 0.50)	0.708
Social functioning	0.17 (0.00, 0.50)	0.17 (0.00, 0.50)	0.283
Emotional wellbeing	0.00 (0.00, 0.33)	0.11 (0.00, 0.44)	0.337
Constipation	0.00 (0.00, 0.50)	0.25 (0.00, 0.75)	0.404
Total score of UCLA GIT 2.0	0.27 (0.16, 0.61)	0.37 (0.13, 0.74)	0.452

Statistically significant results are highlighted in bold font. *Mann-Whitney *U* test, ^#^Chi-square test

*mRSS* modified Rodnan skin score, *FVC* forced vital capacity, *BMI* body mass index;, *Hb* hemoglobin, *ESR* erythrocyte sedimentation rate, *PPI* proton pump inhibitors

We next wanted to analyze whether clinical parameters can be identified that are independently associated with the presence of esophagitis or other pathologic GI tract findings. In multivariable GLMM analysis on the outcome of endoscopic esophagitis, mRSS and EUSTAR reported esophageal symptoms (“reflux and/or dysphagia”) were the only parameters associated with endoscopic esophagitis; however, the associations were very weak (with an OR of only 1.1 for mRSS and a low AUC of 0.61 for esophageal symptoms) (Table 6). Hemoglobin correlated with endoscopic esophagitis in the univariable model, but not in the multivariable model. The UCLA GIT 2.0 total score and its subscales showed no association with endoscopic esophagitis. Similar negative results were obtained in the GLMM analysis for the outcome of pathologic EGD (Table S1 in the online supplement), suggesting that in our real-life cohort, the UCLA GIT 2.0 failed to identify patients with EGD findings.

**Table 6:** Factors associated with esophagitis on EGD (multivariable linear mixed effects models, GLMM)

Multivariable GLMM					
Parameters	Models*	OR	95% CI	***p***	AUC (95%CI)
Age	Model 1	0.98	0.96–1.01	0.289	0.69 (0.60–0.79)
Sex		0.89	0.34–2.31	0.803	
Disease duration		1.00	0.96–1.03	0.925	
PPI		0.50	0.21–1.23	0.133	
**mRSS**	**Model 2**	**1.11**	**1.04–1.18**	**0.003**	**0.76 (0.67–0.84)**
Hb	Model 3	1.33	1.00–1.77	0.051	0.89 (0.83–0.95
Heartburn	Model 4	1.71	0.76–3.83	0.193	0.64 (0.55–0.73)
Regurgitation		1.43	0.59–3.46	0.433	
Dysphagia		1.01	0.46–2.18	0.987	
**Esophageal symptoms**	**Model 5**	**3.25**	**1.00–10.54**	**0.049**	**0.61 (0.52–0.71)**
Stomach symptoms		1.43	0.57–3.60	0.443	
Reflux subscale	Model 6	1.17	0.60–2.26	0.644	0.60 (0.51–0.69)
Distention/bloating subscale	Model 7	0.69	0.43–1.12	0.135	0.66 (0.58–0.75)
Social functioning	Model 8	0.70	0.33–1.50	0.362	0.59 (0.50–0.68)
Emotional wellbeing	Model 9	0.91	0.43–1.95	0.810	0.64 (0.55–0.73)
Total UCLA GIT 2.0 score	Model 10	0.82	0.33–2.02	0.659	0.68 (0.59–0.77)

General linear mixed models. Statistically significant results are highlighted in bold font

*Model 1 contains the covariates age, sex, disease duration and PPI therapy. All other models contain, in addition to the covariates of model 1, the following covariates: model 2: mRSS; model 3: hemoglobin; model 4: the symptoms heartburn, regurgitation, dysphagia, and vomiting; model 5: the symptom clusters “esophageal symptoms” and “stomach symptoms” as per expert opinion; models 6–10: one of the mentioned subscales of UCLA GIT 2.0, respectively the total UCLA GIT 2.0 score

*PPI* proton pump inhibitors, *Hb* hemoglobin, *mRSS* modified Rodnan skin score

## Discussion

To our best knowledge, this is the first study to analyze the performance of the UCLA GIT 2.0 in a large real-life cohort of unselected patients with SSc. Our results show that the UCLA GIT 2.0 score and its reflux subscale identified patients with SSc, in whom EGD was recommended by experts, with a sensitivity of over 70% and a specificity of about 50%.

The recommendation for EGD was made in all patients by a rheumatologist with experience in SSc, at the annual visit of the patient and following a comprehensive investigation, as defined by the EUSTAR guidelines [1]. There was no regular use of the UCLA GIT 2.0 questionnaire to decide further GI tract investigation. We excluded patients with concomitant acute GI bleeding or a history of cancer in the upper GI tract, as in these cases, the indication for EGD would be driven by other criteria than the symptoms captured by the UCLA GIT 2.0.

Considering that several clinical or laboratory data might influence the indication of EGD, or might predict EGD findings, we adjusted the analyses for all these parameters, which were included as covariates for the GLMM models after a careful selection based on clinical judgment and evidence from published literature. For example, we expected the recommendation for EGD to be favored by anemia, possibly caused by gastrointestinal bleeding, which is frequent in SSc, especially in the presence of GAVE [5]; however, our data did not show any association between Hb and the referral to EGD. On the other hand, mRSS was significantly associated with the recommendation for EGD, but the very small OR suggests that this association is of little clinical significance. As expected, patients with a history of Barrett’s esophagus were more frequently referred to EGD. We did not have enough data on significant weight loss or decrease in Hb, and no data on other objective markers of GI involvement, such as F-calprotectin, to include these among the selected covariates.

The recommendation to perform EGD was significantly associated with higher UCLA GIT 2.0 reflux, distention/bloating, and social functioning subscale scores, as well as with higher total scores. As expected, we found similar significant associations for individual symptoms like heartburn, dysphagia, and regurgitation, as well as for these symptoms clustered together as esophageal symptoms and stomach symptoms. These results support the use of the UCLA GIT 2.0 questionnaire in practice, as it provides the attending rheumatologist with detailed information on gastrointestinal symptoms and helps orientating the further investigation of the GI tract.

In the second part of the study, we analyzed the hypothesis that the reflux subscale or the total score of the UCLA GIT 2.0 would be associated with endoscopic esophagitis or with a pathologic EGD in general. Data on the associations of the UCLA GIT 2.0 with objective upper GI tract findings in patients with SSc are scarce. Previous studies analyzed smaller groups of selected patients, in whom GI tract investigation and completion of the UCLA GIT 2.0 were performed systematically and within a narrow time interval [18–20]. A prospective study on 55 patients with SSc and clinically significant upper GI tract symptoms found a moderate correlation between the reflux scale of the UCLA GIT 2.0 with endoscopic esophagitis; the reflux subscale was also discriminative between patients with and without pathologic findings on esophageal manometry [18]. Another study on 40 patients with SSc, of whom 85% reported upper GI tract symptoms, found an association of higher reflux and total UCLA GIT 2.0 scores with decreased amplitude of distal esophageal contractions [19]. A very recent study on 31 patients with SSc, assessing esophageal motility dysfunction by scintigraphy, found a significant association of esophageal emptying activity with the GIT 2.0 reflux score, but not with the other subscales and the total UCLA GIT 2.0 score [20].

In our study on a large cohort of real-life patients, neither the total UCLA GIT 2.0 score nor the reflux subscale correlated with endoscopic esophagitis. The only parameters showing associations with this outcome were the EUSTAR-recorded “esophageal symptoms” (defined as the presence of reflux and/or dysphagia), and the mRSS. For the latter, the very low OR suggests the association is of little clinical importance. Not surprisingly, the symptom interpretation by the physician (as presence or absence of “esophageal symptoms”) performed better than single symptoms such as “heartburn”, as recorded in the patient EMR, in detecting patients with esophagitis.

The lack of correlation between esophagitis and single symptoms or the UCLA GIT 2.0 reflux scale may be explained by several factors, among which the non-systematic use of EGD, the variable time between EGD and the UCLA GIT 2.0 completion, and the use of PPI in about 60% of patients. Moreover, large studies performed by gastroenterologists in patients with gastro-esophageal reflux disease (GERD) have shown that an expert history, as well as GERD questionnaires, such as the reflux disease questionnaire and gastroesophageal reflux disease questionnaire, have important limitations when compared with objective testing for GERD by EGD or functional testing [21–23]. Studies with systematic EGD in unselected patients with SSc are scarce [24, 25]. In a single-center SSc cohort study, Petcu et al. found endoscopic esophagitis in 8/22 patients without any GI symptoms and in 39/57 patients with GI symptoms. Only 12/26 patients with gastroesophageal reflux symptoms had esophagitis on endoscopy [24]. The authors advocate for the routine use of EGD during the early stage of SSc, even in the absence of typical symptoms.

The strengths of our study rely in the large, real-life cohort of unselected patients from a tertiary SSc center with long-standing experience, and in the statistical methods applied, which allow adjusting for a large number of independent parameters potentially associated with the study outcomes. The study also has several limitations, which include the partially retrospective data collection. However, the large majority of the data were collected prospectively following the EUSTAR recommendations [1]. There was considerable variability in performing EGD, as in some patients this was not done despite being recommended, and in others it may have been done in another center, with the results not recorded in the EMR of our hospital. However, over 70% of EGD recommended by our center were performed and the respective results were available in the hospital EMR. It is possible that some reports of EGD performed outside our hospital may have not reached us, but we assume that in many of these cases EGD was probably not done, as our center strives to obtain all medical information of the patients and communication between local medical facilities is generally good. Another limitation is the time of ± 3 months allowed between questionnaire completion and EGD, which is quite long and may have contributed to the lack of correlation between UCLA GIT 2.0 scores and the results of the EGD. Finally, yet importantly, treatment with PPI was not recorded into detail and we were not able to analyze the indication for PPI, doses, or compliance.

## Conclusions

In a large real-life cohort of unselected patients with SSc, we found a significant association of the UCLA GIT 2.0 score with the interpretation of GI symptoms by rheumatologists and consecutive recommendations for EGD. However, there was no association between the UCLA GIT 2.0 score, or its subscales, with endoscopic esophagitis, nor with any pathologic findings on EGD. Even the correlation between single symptoms, such as heartburn and dysphagia, and endoscopic esophagitis, was poor. We conclude that, while using the UCLA GIT 2.0 in the routine care of patients with SSc may help the rheumatologist to better understand the burden of GI symptoms in the individual patient, it should not be used as a stand-alone instrument to identify an indication of EGD. The question of whether all or selected patients with SSc should be investigated by EGD needs to be addressed by further studies.

## Supplementary Information

**Additional file 1.**

## Data Availability

Data are available from the authors, upon request.

## Abbreviations

AUC: Area under the curve
BMI: Body mass index
EGD: Esophago-gastro-duodenoscopy
EMR: Electronic medical records
ESR: Erythrocyte sedimentation rate
EUSTAR: European Scleroderma Trials and Research Group
FVC: Forced vital capacity
GAVE: Gastric antral vascular ectasia
GI: Gastrointestinal
GLMM: Multivariable generalized linear mixed effects model
Hb: Hemoglobin
HRQoL: Health-related quality of life
mRSS: Modified Rodnan skin score
Q1, Q3: Interquartile range
OR: Odds ratio
PPI: Proton pump inhibitors
ROC curve analysis: Receiver operating characteristic curve analysis
SSc: Systemic sclerosis
UCLA GIT 2.0: University of California Los Angeles Scleroderma Clinical Trial Consortium Gastrointestinal Tract Instrument 2.0
95% CI: 95% confidence interval
