# Supplementary material for: Performance of the UCLA Scleroderma Clinical Trials Consortium Gastrointestinal Tract 2.0 instrument as a clinical decision aid in the routine clinical care of patients with systemic sclerosis
Source: Arthritis Res Ther. 2021 Apr 22;23:125. doi: 10.1186/s13075-021-02506-x (PMC8061014; doi:10.1186/s13075-021-02506-x)
Supplement: Supplementary file 1 — Additional file 1. [file 13075_2021_2506_MOESM1_ESM.docx]

Symptom definition

Data on single symptoms were obtained by history and recorded by rheumatologists in the

medical charts (heartburn, de4ned as a burning feeling in the chest caused by stomach acid

travelling up towards the throat). Data from the EUSTAR database (esophageal, stomach and

intes'nal symptoms) are the results on interpreta'on of symptoms by the inves'gator. They

are de4ned in the database as follows: esophageal symptoms by dysphagia and/or re<ux,

stomach symptoms by early sa'ety and/or vomi'ng, and intes'nal symptoms by diarrhea,

bloa'ng, and/or cons'pa'on. In the study database, all these data are dichotomous

(present/absent)

Data on single symptoms were obtained by history and recorded by rheumatologists in the

medical charts (heartburn, de4ned as a burning feeling in the chest caused by stomach acid

travelling up towards the throat). Data from the EUSTAR database (esophageal, stomach and

intes'nal symptoms) are the results on interpreta'on of symptoms by the inves'gator. They

are de4ned in the database as follows: esophageal symptoms by dysphagia and/or re<ux,

stomach symptoms by early sa'ety and/or vomi'ng, and intes'nal symptoms by diarrhea,

bloa'ng, and/or cons'pa'on. In the study database, all these data are dichotomous

(present/absent)

Data on single symptoms were obtained by history and recorded by rheumatologists in the

medical charts (heartburn, de4ned as a burning feeling in the chest caused by stomach acid

travelling up towards the throat). Data from the EUSTAR database (esophageal, stomach and

intes'nal symptoms) are the results on interpreta'on of symptoms by the inves'gator. They

are de4ned in the database as follows: esophageal symptoms by dysphagia and/or re<ux,

stomach symptoms by early sa'ety and/or vomi'ng, and intes'nal symptoms by diarrhea,

bloa'ng, and/or cons'pa'on. In the study database, all these data are dichotomous

(present/absent)

Data on single symptoms were obtained by history and recorded by rheumatologists in the medical charts (e.g.: heartburn, defined as a burning feeling in the chest caused by stomach acid travelling up towards the throat).

Data from the EUSTAR database (esophageal, stomach and intestinal symptoms) are the results on interpretation of symptoms by the investigator. They are defined in the database as follows: esophageal symptoms by dysphagia and/or reflux, stomach symptoms by early satiety and/or vomiting, and intestinal symptoms by diarrhea, bloating, and/or constipation. In the study database, all these data are dichotomous (present/absent)

Figure S1. Selection and distribution of patient visits (study outcome: referral to EGD)

EGD=esophagogastroduodenoscopy

Figure S2- Selection and distribution of patient visits with EGD (study outcome: detection of esophagitis on EGD, detection of any pathologic finding on EGD)

Table S1. Factors associated with abnormal/pathologic EGD (multivariable linear mixed effects models, GLMM).

| Multivariable GLMM | | | | |  |
| --- | --- | --- | --- | --- | --- |
| Parameters | **Models*** | **OR** | **95% CI** | **p** | **AUC (95% CI)** |
| Age | Model 1 | 1.02 | 0.99 – 1.05 | 0.235 | 0.94 (0.91-0.97) |
| Sex |  | 0.91 | 0.35 – 2.37 | 0.848 |  |
| Disease duration |  | 1.00 | 0.97 – 1.04 | 1.000 |  |
| PPI |  | 1.87 | 0.88 – 3.98 | 0.103 |  |
| **mRSS** | **Model 2** | **1.08** | **1.00 – 1.15** | **0.045** | **0.93 (0.89-0.97)** |
| Hb | Model 3 | 0.97 | 0.75 – 1.26 | 0.827 | 0.98 (0.96-0.99) |
| **Heartburn** | **Model 4** | **3.38** | **1.32 – 8.68** | **0.011** | **0.92 (0.88-0.96)** |
| Regurgitation |  | 0.69 | 0.25 – 1.89 | 0.470 |  |
| Dysphagia |  | 0.73 | 0.32 – 1.65 | 0.449 |  |
| Esophageal symptoms | Model 5 | 1.49 | 0.63 – 3.49 | **0.365** | **0.96 (0.93-0.98)** |
| Stomach symptoms |  | 0.82 | 0.35 – 1.93 | 0.647 |  |
| Reflux subscale | Model 6 | 1.41 | 0.72 – 2.75 | 0.313 | 0.92 (0.88-0.95) |
| **Distention/bloating subscale** | **Model 7** | **0.70** | **0.69 – 0.70** | **<0.001** | **0.94 (0.91-0.97)** |
| Social functioning | Model 8 | 1.07 | 0.51 – 2.26 | 0.853 | 0.94 (0.91-0.97) |
| Emotional wellbeing | Model 9 | 1.13 | 0.54 – 2.37 | 0.737 | 0.92 (0.88-0.95) |
| Total score UCLA GIT 2.0 | Model 10 | 1.02 | 0.42 – 2.46 | 0972 | 0.87 (0.82-0.92) |

General linear mixed models. Statistically significant results are highlighted in bold font

* Model 1 contains the covariates age, sex, disease duration and PPI therapy. All other models contain, in addition to the covariates of model 1, the following covariates: Model 2: mRSS; Model 3: hemoglobin; Model 4: the symptoms heartburn, regurgitation, dysphagia and vomiting. Model 5: the symptom clusters “esophageal symptoms” and “stomach symptoms” as per expert opinion. Models 6-10: one of the mentioned subscales of UCLA GIT 2.0, respectively the total UCLA GIT 2.0 score.

PPI= proton pump inhibitors; Hb=haemoglobin; mRSSS=modified Rodnan skin score
